# Histone crotonylation-centric gene regulation

**DOI:** 10.1186/s13072-021-00385-9

**Published:** 2021-02-06

**Authors:** Kun Li, Ziqiang Wang

**Affiliations:** 1 Department of Nuclear Medicine, The First Affiliated Hospital of Shandong First Medical University, Jinan, 250014 China; 2 Medical Research Center, The First Affiliated Hospital of Shandong First Medical University, Jinan, 250014 China; 3 Biomedical Sciences College & Shandong Medicinal Biotechnology Centre, Shandong First Medical University & Shandong Academy of Medical Sciences, Jinan, 250062 China

**Keywords:** Histone crotonylation, Gene regulation, Writer, Eraser, Reader

## Abstract

Histone crotonylation is a recently described post-translational modification that occurs at multiple identified histone lysine crotonylation sites. An increasing number of studies have demonstrated that histone crotonylation at DNA regulatory elements plays an important role in the activation of gene transcription. However, among others, we have shown that elevated cellular crotonylation levels result in the inhibition of endocytosis-related gene expression and pro-growth gene expression, implicating the complexity of histone crotonylation in gene regulation. Therefore, it is important to understand how histone crotonylation is regulated and how it, in turn, regulates the expression of its target genes. In this review, we summarize the regulatory factors that control histone crotonylation and discuss the role of different histone crotonylation sites in regulating gene expression, while providing novel insights into the central role of histone crotonylation in gene regulation.

## Background

Lysine crotonylation is a histone modification first described in 2011 [1]. To date, multiple histone and non-histone lysine crotonylation (Kcr) sites have been identified in various organisms [2–7]. The majority of histone Kcr is enriched in transcriptional start sites (TSSs) and enhancer regions, suggesting a potential role for histone Kcr in gene regulation [1]. Most studies have reported that histone Kcr at the gene promoter facilitates the transcription of genes. However, we, as well as others, have shown that increased cellular crotonylation levels inhibit endocytosis-related and pro-growth gene expression [8, 9].

Increasing evidence has demonstrated that histone Kcr is associated with physiological and pathological processes, such as differentiation [1, 10], tissue injury [11], virus infection [12, 13], tumorigenesis [14], and neurodegenerative disease [8]. The first histone Kcr-related biological process to be discovered was germ cell differentiation. During spermatogenesis, histone Kcr is enriched in promoters of highly expressed testes genes, including a number of X-linked genes that function to maintain sex chromosome activation in haploid cells. This results in the differentiation of male germinal cells immediately following meiosis [1]. Histone Kcr was next found to be related to nephropathy, including acute kidney injury (AKI). Researchers found that increased histone crotonylation prevented AKI and a decrease in renal function via increasing PGC-1α and sirtuin-3 levels and decreasing CCL2 expression [11].

In addition, histone Kcr functions as an inducer for the reactivation of latent human immunodeficiency virus (HIV) by promoting viral gene transcription via the HIV long-terminal repeat (LTR) [12]. Moreover, histone Kcr expression is altered in a number of cancers, including liver, stomach, kidney, thyroid, esophagus, colon, pancreas and lung carcinomas. In hepatocellular carcinoma (HCC), the induction of Kcr through siRNAs targeting histone deacetylases (HDACs) or HDAC inhibitors inhibits hepatoma cell motility and proliferation [14]. Our recent study showed that induction of histone crotonylation inhibits the expression of endocytosis-related genes, which are important for Aβ uptake during the development of Alzheimer’s disease (AD), through decreasing levels of histone acetylation in the promoters of these genes, suggesting that histone crotonylation is a potential therapeutic target against AD [8].

In this review, we summarize and discuss the molecules and regulatory patterns that modulate histone crotonylation. These molecules mainly function as crotonyltransferases, decrotonyltransferases, and readers. We also summarize and discuss the different roles of specific histone crotonylation in gene regulation.

## Factors regulating histone crotonylation

Several factors are associated with histone crotonylation-mediated gene regulation. They function as writers, erasers, readers, or regulators for histone crotonylation (Table 1).

**Table 1:** The regulatory factors for histone crotonylation

Factor	Regulatory pattern	Crotonylation site	References
P300	Writer	H3K18	[15]
MOF		H3K4, H3K9, H3K18, H3K23, H4K8, and H4K12	[17]
GCN5		H3K9, H3K14, H3K18, H3K23, and H3K27	[18]
Esa1		H4K5, H4K8, H4K12, and H4K16	[18]
SIRT 1, 2, 3	Eraser	H3K4	[19]
HDAC1, 2, 3		H3K4, H3K9, H3K23, H4K8, H4K12 (HDAC1), and H3K23 (HDAC2, 3)	[20]
Taf14	Reader	H3K9	[26]
YEATS2		H3K27	[27]
AF9		H3K9, H3K18 and H3K27	[28]
MOZ and DPF2		H3K14	[29]
NEAT1	Regulator with unknown mechanism	H3K27	[8]
CDYL		H2BK12, H3K9, H3K27, and H4K8	[10]
ACSS2		H3K4 and H3K18	[12, 15]
RNF8		/	[33]

### Writers

P300 was the first histone crotonyltransferase identified. Researchers found that only p300 showed measurable histone crotonyltransferase (HCT) activity relative to other histone acetyltransferases (HATs) including GCN5, TIP60, and MOF. Additionally, in vitro experiments showed that knockdown of p300 or its paralog CBP via siRNAs reduced the global levels of H3K18Cr [15]. However, compared to its acetyltransferase activity, the crotonyltransferase activity of p300 showed a nearly 62-fold decrease due to the restricted size of an aliphatic back pocket of p300 [16], suggesting other co-factors are required to enhance the catalytic efficiency of p300.

In addition, other reports have demonstrated that MOFs can also catalyze histone H3 crotonylation at lysine residues 4, 9, 18, and 23 and histone H4 at lysine 8 and 12 in HeLa cells [17], as well as that GCN5 can target lysine residues at positions 9, 14, 18, 23, and 27 in histone H3 for crotonylation in budding yeast [18]. In addition, Esa1, the homolog of human MOF, has been shown to catalyze crotonylation at lysine residues 5, 8, 12, and 16 in histone H4 [18].

### Erasers

The first discovered histone decrotonylases were members of the sirtuin family of class III histone deacetylases: Sirt1, Sirt2, and Sirt3. Using a chemical proteomics approach, these three deacetylases were found to recognize the histone H3K4Cr mark by directly binding to the crotonylated histone peptide via a π–π interaction, indicating that they functioned as “erasers” of crotonylation, removing Kcr marks from histone proteins [19].

HDAC1, HDAC2, and HDAC3, members of the class I histone deacetylases, also mediate histone decrotonylation. Compared to class III histone deacetylases, class I histone deacetylases possess a greater regulatory effect on histone decrotonylation. HDAC1 is active at multiple crotonylated sites, including H3K4, H3K9, H3K23, H4K8, and H4K12, whereas HDAC2 and 3 are active at H3K23Cr; however, SIRT1 is the only eraser responsible for removing Kcr at H3K9Cr and H4K8Cr [20].

### Readers

Recognition of histone modifications by “reader” modules constitutes a major mechanism of epigenetic regulation. The first discovered effective reader of histone crotonylation was the YEATS domain, which is an evolutionarily conserved gene regulatory factor found in species from human to yeast. YEATS family members can form a variety of important complexes that participate in transcriptional regulation, histone modification, histone deposition, and chromatin remodeling [21]. To date, several proteins have been reported to contain the YEATS domain, such as the transcription factor complexes TFIID and TFIIF, chromatin-remodeling complexes INO80, SWI/SNF and RSC, and the histone acetyltransferase complex NuA3 [22–25]. The Taf14 YEATS domain was found to recognize H3K9Cr by adopting an immunoglobin-like β sandwich fold containing eight anti-parallel β strands linked by short loops that form a binding site for H3K9Cr [26]. The YEATS domain of YEATS2 has the strongest affinity for H3K27Cr with lower binding efficiencies for H4K4Cr, H3K12Cr, H3K23Cr, and H3K9Cr via an end-open aromatic sandwich pocket for Kcr binding [27]. The AF9 YEATS binds strongly to H3K9Cr, H3K18Cr, and H3K27Cr via a pocket formed by the L1, L4, and L6 loops of AF9 [28].

The second discovered histone crotonylation reader was the double PHD finger (DPF) domain. The DPF domains of human MOZ (also known as KAT6A) and DPF2 (also known as BAF45d), two histone acetylation-binding proteins, recognize H3K14Cr via a hydrophobic “dead-end” pocket [29].

### Other regulators

In our study on the role of the long non-coding RNAs in AD, we found that NEAT1 is associated with the acetyltransferase P300/CBP complex and that knockdown of NEAT1 increases histone Kcr and decreases acetylation at H3K27, suggesting that NEAT1 mediates histone acetylation and inhibits histone crotonylation at the same lysine sites [8].

Ring finger protein 8 (RNF8), a ubiquitin ligase (E3), has roles in the DNA damage response [30, 31] and cell cycle progression [32]. A study to determine the role of RNF8 in the development of spermatids found that RNF8 epigenetically regulates a set of sex-linked genes that tend to escape post-meiotic silencing and is activated in round spermatids through increased lysine crotonylated histone around TSSs of these genes, and thereby, an alteration of chromatin conformation. This suggests RNF8 acts as an important regulator of spermatogenesis through epigenetic programming in sex chromosomes [33].

In addition, acyl-CoA synthetase short-chain family member 2 (ACSS2) was found to promote the crotonylation at H3K4 and H3K18 by increasing the cellular levels of crotonyl-CoA [12, 15]. Chromodomain Y-like (CDYL), the chromodomain Y-like transcription corepressor, acts as a crotonyl-CoA hydratase and inhibits histone crotonylation at H2BK12, H3K9, H3K27, and H4K8 through its chromodomain and CoAP domains [10].

## The regulatory role of histone crotonylation in gene expression

Like other histone modifications, the main function of histone crotonylation is to regulate gene expression. In this section, we summarized and discussed the regulatory role of histone crotonylation in gene expression (Table 2).

**Table 2:** The regulatory role of histone crotonylation in gene expression

Histone crotonylation	Target	Regulatory role	References
Histone Kcr	PGC-1α and sirtuin-3	Promoting transcription	[11]
	Ptk2, Tshz3, Wapal	Promoting transcription	[19]
H2BK12Cr	Pin4, Ccdc160, Tceal, Rnf138rt1, and 4933436I01Rik	Promoting transcription	[10]
H3K4Cr	HIV LTR	Promoting transcription	[35]
H3K9Cr	Pro-growth genes	Inhibiting transcription	[9]
H3K18Cr	Il6, Gbp2, Ifit1, and Rsad2	Promoting transcription	[15]
	RANDP3L, AOX1, GRPR, and NCAM1	Promoting transcription	[20]
	HIV long-terminal repeat (LTR)	Promoting transcription	[35]
H3K27Cr	Endocytosis-related genes	Inhibiting transcription	[8]

### Transcriptional promotion

The first evidence of histone Kcr regulating gene expression was found in male germinal cells immediately following meiosis. Kcr is enriched on sex chromosomes and specifically marks testis-specific genes, including a significant proportion of X-linked genes that escape sex chromosome inactivation in haploid cells [1, 34]. Then, an investigation to identify “erasers” of histone Kcr found that Sirt3 decreased the expression levels of Ptk2, Tshz3, and Wapal, as well as the enrichment of crotonylated histone at the transcription stat sites of these target genes, suggesting that histone crotonylation might function as a positive regulator of the expression of these genes [19]. In addition, during AKI, histone crotonylation in kidney tissue increases, which protects the kidney from AKI by increasing the expression of the mitochondrial biogenesis regulator PGC-1α and the sirtuin-3 decrotonylase by increasing the enrichment of histone crotonylation at these genes [11].

Specifically, several histone Kcr sites have been associated with gene activation, including H3K18Cr for “de novo-activated” genes such as Il6, Gbp2, Ifit1, and Rsad2 [15]; H3K18Cr for RANDP3L, AOX1, GRPR, and NCAM1 [20]; H2BK12Cr for post-meiotic genes that play an important role in spermatogenesis [10]; and H3K4Cr and H3K18Cr for the HIV LTR, a key regulator for the establishment of latent reservoirs [35] for the promotion of LTR transcription and the reactivation of HIV from latency [12].

### Transcriptional repression

Our previous study investigated the role of NEAT1, a long non-coding RNA that functions as an important regulator of gene expression [36, 37], in the development of AD. We found that NEAT1 knockdown increases global H3K27Cr and decreases global H3K27Ac levels. Further, exogenous addition of crotonic acid decreased the expression levels of endocytosis-related genes, including CAV2, TGFB2, and TGFBR1 through increasing H3K27Cr and decreasing H3K27Ac at these gene promoters [8]. This suggests that H3K27Cr functions as a marker of transcriptional repression of endocytosis-related genes.

In addition, a recent study illustrating the links between metabolic state and gene expression found that H3K9 crotonylation, which peaks at pro-growth genes, results in gene repression, indicating that H3K9 crotonylation is associated with transcriptional repression of pro-growth genes [9].

## Conclusion and perspective

**Fig. 1:**
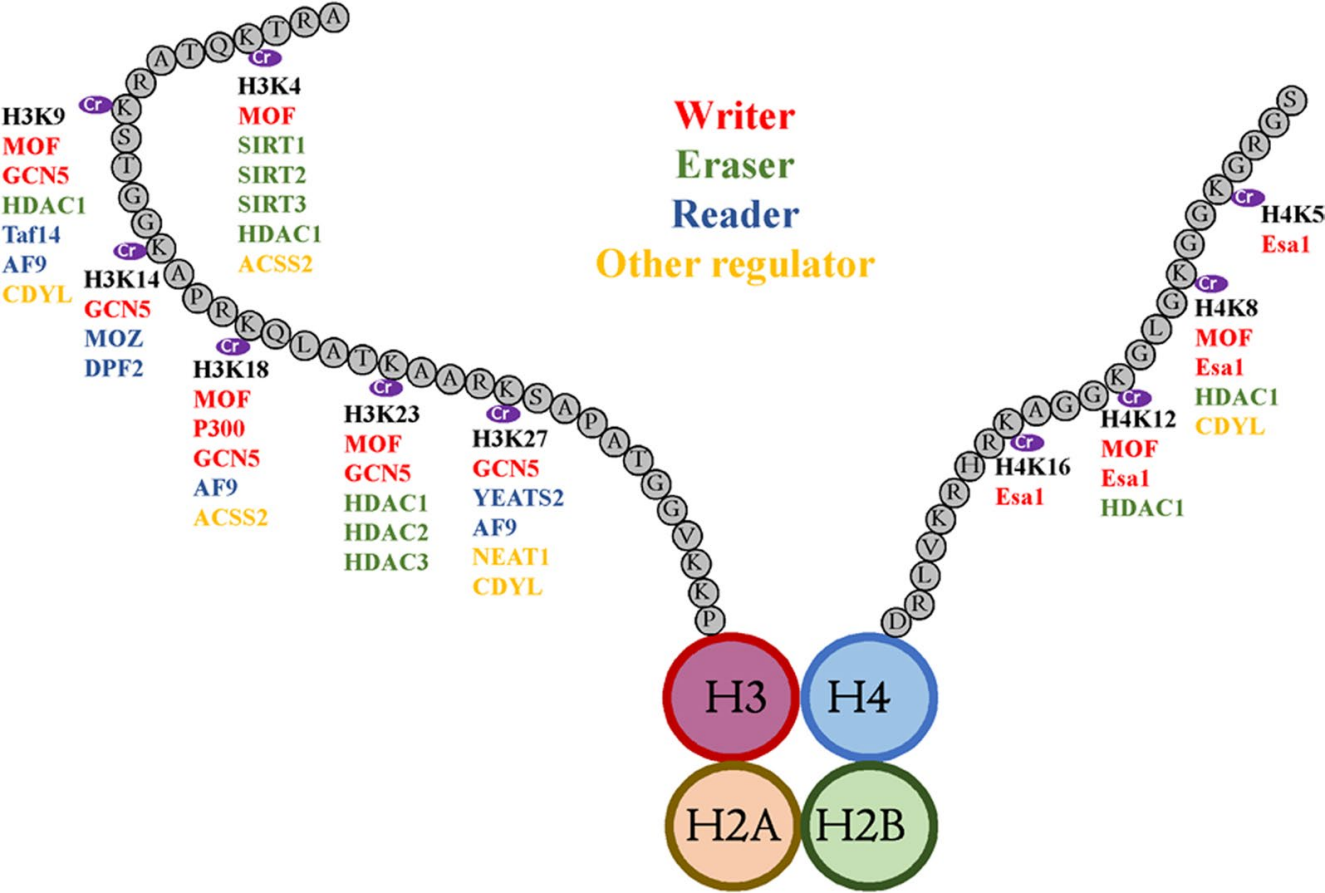
Histone crotonylation and its regulating factors. Schematic model shows the principal lysine crotonylation sites on histones H3 and H4 and the reported writers (crotonyltransferase), erasers (decrotonylase), readers, and other regulators for each lysine crotonylation

Histone crotonylation is a novel histone modification that participates in multiple biological and pathological processes. In this review, we summarized the factors, along with their regulatory patterns, which regulate histone crotonylation and are involved in histone crotonylation-mediated gene regulation. Moreover, crotonyltransferases, decrotonyltransferases, transcription factors, and regulators with unknown mechanisms participate in histone Kcr (Fig. 1). In addition, we discussed the role of histone crotonylation in gene regulation, highlighting that histone crotonylation functions as both an activator and repressor of gene transcription.

Overall, this review provides novel insights into histone crotonylation-centric gene regulation and highlights its potential therapeutic targets for the treatment of human diseases, although the underlying molecular mechanisms still require further clarification. Specifically, to date, no single active or repressive TSS has been evidenced marked only with crotonylation and different histone acylations, such as crotonylation, acetylation, propionylation and butyrylation, seem to coordinate to regulate gene transcription [1, 38, 39]. Therefore, more approaches are needed in the future to characterize the role of histone crotonylation in gene regulation. Since a number of writers, readers and erasers for crotonylation were identified, there is a high chance that more specific regulators will have a major impact on understanding the interplay between crotonylation and other acylations, and the role of specific histone crotonylation sites in gene transcription.

## Data Availability

Not applicable.

## Abbreviations

Kcr: Lysine crotonylation
TSSs: Transcriptional start sites
AKI: Acute kidney injury
HIV: Human immunodeficiency virus
LTR: Long-terminal repeat
HCC: Hepatocellular carcinoma
HDACs: Histone deacetylases
AD: Alzheimer’s disease
HCT: Histone crotonyltransferase
HATs: Histone acetyltransferases
DPF: Double PHD finger
RNF8: Ring finger protein 8
ACSS2: Acyl-CoA synthetase short-chain family member 2
CDYL: Chromodomain Y-like
